# Efficacy and Safety of Astaxanthin in the Management of Oral Submucous Fibrosis: A Preliminary Randomized Controlled Trial

**DOI:** 10.7759/cureus.54667

**Published:** 2024-02-22

**Authors:** Elumalai Perumal, Santosh Patil, Mohmed Isaqali Karobari

**Affiliations:** 1 Center for Global Health Research, Saveetha Medical College and Hospitals, Saveetha Institute of Medical and Technical Sciences, Chennai, IND

**Keywords:** carotenoid, burning sensation, mouth opening, anti-inflammatory, randomized controlled trial, oral submucous fibrosis, astaxanthin

## Abstract

Background: Oral submucous fibrosis (OSMF) is a chronic, potentially malignant disorder characterized by progressive fibrosis of the oral mucosa, leading to restricted mouth opening and discomfort. This study investigates the efficacy and safety of astaxanthin, a potent antioxidant and anti-inflammatory carotenoid, in the comprehensive management of OSMF.

Methods: A randomized, double-blind, placebo-controlled trial was conducted with 68 eligible participants diagnosed with OSMF. Participants were randomly assigned to the experimental group (astaxanthin capsules, 5 mg twice daily) or the control group (placebo capsules) for 12 weeks. Primary outcomes included changes in mouth opening and burning sensation assessed by Visual Analog Scale (VAS). Adverse events were monitored to evaluate safety.

Results: The experimental group demonstrated a statistically significant improvement in mouth opening compared to the control group over the 12-week intervention (p < 0.001). Additionally, the experimental group reported a significant reduction in burning sensation, as indicated by VAS scores (p < 0.001). Adverse events were generally mild and comparable between groups.

Conclusion: This study suggests that astaxanthin may have a positive impact on mouth opening and burning sensation in individuals with OSMF. The safety profile observed supports the feasibility of astaxanthin as a potential therapeutic adjunct in OSMF management. Further research with larger sample sizes and extended follow-up periods is warranted to validate these findings.

## Introduction

Oral submucous fibrosis (OSMF) is a chronic and potentially malignant disorder of the oral cavity characterized by progressive fibrosis of the submucosal tissues [1]. It primarily affects the oral mucosa, leading to restricted mouth opening, burning sensations, and significant discomfort for affected individuals [2]. The condition is associated with a range of risk factors, including areca nut and betel quid chewing, with a high prevalence in regions where these habits are prevalent [3].

Despite its well-documented association with these habits, the precise etiology of OSMF remains complex and multifactorial, involving genetic, immunologic, and environmental factors [4]. Current therapeutic approaches primarily focus on symptom management, with limited success in halting or reversing the fibrotic progression of the disease [3,5]. Therefore, there is a critical need for innovative interventions to address the underlying pathophysiology of OSMF and offer more effective therapeutic outcomes. Astaxanthin, a naturally occurring carotenoid, has gained attention for its potent antioxidant and anti-inflammatory properties [6]. Derived from microalgae and seafood, astaxanthin has demonstrated diverse health benefits, ranging from cardiovascular protection to immune modulation [7]. Its unique molecular structure allows it to quench reactive oxygen species (ROS) and modulate inflammatory pathways, making it a promising candidate for conditions characterized by oxidative stress and inflammation, such as OSMF [8].

Studies have provided evidence of astaxanthin's anti-fibrotic effects, suggesting its potential to mitigate the pathological processes leading to tissue fibrosis [9]. Astaxanthin's ability to modulate the transforming growth factor-β1 (TGF-β1) signaling pathway, a key player in fibrosis, further supports its candidacy as a therapeutic agent in fibrotic disorders [9]. Given the global burden of OSMF and its impact on oral health and quality of life, identifying effective interventions is of paramount importance. If astaxanthin proves efficacious in improving mouth opening, alleviating burning sensations, and demonstrating a favorable safety profile, it could represent a breakthrough in OSMF management. This study contributes to the evolving landscape of therapeutic options for OSMF, offering a potential avenue for more targeted and efficacious interventions in the future.

## Materials and methods

Study design

A meticulously crafted and methodically executed randomized, double-blind, placebo-controlled trial was undertaken to investigate the efficacy and safety of astaxanthin in the comprehensive management of oral submucous fibrosis (OSMF). The study was intentionally structured to enroll a precise and representative cohort comprising 68 eligible participants, ensuring a comprehensive exploration of the intervention's impact on OSMF.

Ethical considerations

Informed consent was obtained from all participants and the study protocol adhered to the principles outlined in the Declaration of Helsinki. Ethical approval was granted by the Institutional Review Board, Saveetha Medical College and Hospitals, Saveetha Institute of Medical and Technical Sciences, Chennai, India, with an ethical approval code (074/01/2024/Faculty/SRB/SMCH).

Participant recruitment

Between the defined period from June 2022 and May 2023, a thorough screening process meticulously assessed potential participants against stringent inclusion and exclusion criteria. A total of 68 eligible individuals, aged between 18 and 60 years, clinically diagnosed with OSMF, and providing voluntary informed consent, were systematically enrolled in the study. The inclusion and exclusion criteria were carefully designed to ensure the study's relevance to the target population while excluding confounding variables.

Inclusion Criteria

The patients who visited the Saveetha Medical College and Hospital outpatient department were included in the current study. Patients aged between 18 and 60 years who were clinically diagnosed with oral submucous fibrosis and willing to provide informed consent were included in the current study.

Exclusion Criteria

Patients with pregnancy or lactation, known allergy to astaxanthin, presence of severe systemic conditions and illnesses, and concurrent participation in other clinical trials were excluded from the current study.

Randomization and blinding

Employing a robust randomization process, participants were assigned randomly to either the experimental or control group using a computer-generated sequence. To maintain the study's integrity, both investigators and participants were meticulously kept blind to the treatment assignments. The blinding process was further reinforced by dispensing identical-looking capsules, indistinguishably containing either astaxanthin (5 mg) or a precisely matched placebo. This stringent blinding methodology aimed to eliminate potential biases and enhance the internal validity of the study.

Intervention

The intervention phase of the study was meticulously executed with a detailed focus on both the experimental and control groups. The goal was to assess the impact of astaxanthin on oral submucous fibrosis (OSMF) over a 12-week period.

Experimental Group

Participants allocated to the experimental group were administered astaxanthin, HealthVit Tablet 4mg (2 Tablets BID after food) for the entirety of the 12-week intervention period. The dosage selection was based on existing literature and aimed to ascertain the potential therapeutic effects of astaxanthin used to reduce pain and inflammation [10]. The meticulous choice of a twice-daily regimen aimed to ensure a sustained presence of astaxanthin in the participants' system, allowing for a comprehensive evaluation of its efficacy over time.

Control Group

Participants assigned to the control group received placebo capsules, indistinguishable from the astaxanthin capsules, twice daily for the same 12-week duration. The use of a placebo control allowed for the isolation of astaxanthin's specific effects from potential placebo effects, contributing to a more robust assessment of its impact on OSMF. The placebo capsules were carefully formulated to match the appearance and texture of the astaxanthin capsules, ensuring a double-blinded approach to treatment. This approach aimed to enhance the reliability of the study's findings by minimizing bias associated with participant and investigator expectations.

Outcome measures

The assessment of the study's outcomes was conducted with meticulous attention to detail, with a primary focus on key parameters related to oral submucous fibrosis (OSMF). The primary outcome measures were designed to comprehensively capture the impact of astaxanthin on OSMF, utilizing both objective clinical measures and subjective patient-reported experiences.

Mouth Opening

The primary objective was to quantify changes in mouth opening, a pivotal indicator of OSMF severity. Baseline measurements were established during the initial assessment, and subsequent measurements were taken at 4, 8, and 12 weeks. Interincisal mouth opening for each participant was quantified utilizing a digital Vernier caliper, a precise measuring instrument.

Pain/Burning Sensation as Assessed by VAS

The intensity of the burning sensation was documented using a Visual Analog Scale (VAS) ranging from 0 to 10, with a higher score indicating more severe discomfort. This patient-reported outcome was systematically gathered at regular intervals during the 12-week intervention period, providing valuable insights into the subjective influence of astaxanthin on symptomatology [11].

Adverse Events

During the 12-week intervention period, vigilant monitoring of adverse events and safety parameters was systematically conducted to ensure a comprehensive assessment of the participants' well-being. Adverse events, encompassing any untoward medical occurrences, were actively solicited from the participants and recorded in a structured manner.

Data collection

Baseline assessments were conducted, including clinical examination, severity scoring, and symptom evaluation. Follow-up assessments were performed at 4, 8, and 12 weeks to monitor changes and gather relevant data.

Statistical analysis

Statistical analyses for this study were performed using IBM SPSS Statistics version 23.0 (IBM Corp., Armonk, NY, USA) by applying t-tests, chi-square tests, and intent-to-treat analysis with a significance level of 0.05. Employed subgroup analyses to explore treatment response variations.

## Results

Demographic characteristics

The study ensured a balanced distribution of participants between the experimental and control groups, comprising 68 eligible participants with comparable baseline characteristics. Age, gender distribution, and the severity of oral submucous fibrosis (OSMF) were well-matched, minimizing potential confounding factors (Table 1).

**Table 1:** Demographic characteristics of the participants Oral submucous fibrosis - OSMF

Characteristic	Experimental Group (n=34)	Control Group (n=34)
Age (mean ± SD)	42 ± 6	41 ± 7
Gender (Male/Female)	18/16	19/15
OSMF Severity (mild/moderate/severe)	10/18/6	12/16/6

Baseline characteristics

At baseline, the experimental group exhibited a slightly lower mean mouth opening (26 mm) compared to the control group (27 mm). The Visual Analog Scale (VAS) for burning sensation indicated a slightly higher score in the experimental group (7) compared to the control group (6.5). These baseline measures laid the foundation for evaluating changes throughout the intervention (Table 2).

**Table 2:** Baseline characteristics of participants Visual Analog Scale -VAS

Characteristic	Experimental Group	Control Group
Mouth Opening (mm) - Mean ± SD	26 ± 4	27 ± 3
VAS for Burning Sensation - Mean ± SD	7 ± 1.5	6.5 ± 1.2

Primary outcome: mouth opening

Over the 12-week intervention period, the experimental group demonstrated a progressive and statistically significant improvement in mouth opening compared to the control group. By the study's conclusion, the experimental group exhibited a substantial increase, with a mean mouth opening of 32 mm, while the control group remained at 27 mm (p < 0.001). This suggests a significant positive effect of astaxanthin on increasing mouth opening in individuals with OSMF (Table 3).

**Table 3:** Mouth opening of participants The asterisk (*) denotes statistical significance p<0.05 between the control and experimental groups.

Time Point (weeks)	Experimental Group (mean ± SD)	Control Group (mean ± SD)	p-value
Baseline	26 ± 4	27 ± 3	
4	28 ± 3	27.5 ± 2.5	0.12
8	30 ± 2.5	28 ± 2	0.03*
12	32 ± 2	27 ± 2	<0.001*

VAS for burning sensation

The experimental group exhibited a significant reduction in burning sensation over the 12 weeks, as evidenced by decreasing VAS scores. At 12 weeks, the experimental group reported a mean VAS score of 3, whereas the control group maintained a higher mean score of 5.5 (p < 0.001). This indicates a significant therapeutic effect of astaxanthin in alleviating the burning sensation associated with OSMF (Table 4).

**Table 4:** Visual analog scale for burning sensation of participants The asterisk (*) denotes statistical significance p<0.05 between the control and experimental groups.

Time Point (weeks)	Experimental Group (mean ± SD)	Control Group (mean ± SD)	p-value
Baseline	7 ± 1.5	6.5 ± 1.2	
4	5.5 ± 1	6 ± 1	0.08
8	4 ± 1	5 ± 1	0.001*
12	3 ± 0.5	5.5 ± 1	<0.001*

Adverse events

Adverse events were generally mild and comparable between groups. The most commonly reported event was mild gastrointestinal upset. While the experimental group reported a slightly higher incidence of adverse events, the overall safety profile appeared acceptable. The study suggests that astaxanthin is well-tolerated, with adverse events mainly consisting of mild and expected gastrointestinal symptoms (Table 5).

**Table 5:** Adverse events recorded in participants

Adverse Event	Experimental Group (n)	Control Group (n)
Mild gastrointestinal upset	2	1
Allergic reaction to placebo	0	1
Headache	1	2

## Discussion

Oral submucous fibrosis (OSMF) is a chronic, potentially malignant disorder characterized by progressive fibrosis of the oral mucosa. The present randomized, double-blind, placebo-controlled trial aimed to investigate the efficacy and safety of astaxanthin in the comprehensive management of OSMF. The study, meticulously designed and executed, provides valuable insights into the potential therapeutic role of astaxanthin in addressing the clinical manifestations of OSMF.

One of the primary outcomes assessed in this study was the improvement in mouth opening, a critical parameter reflecting the severity of OSMF. The findings demonstrated a statistically significant increase in mouth opening in the experimental group compared to the control group over the 12-week intervention period. This outcome aligns with existing literature on the anti-fibrotic properties of astaxanthin [9]. Astaxanthin, a potent antioxidant derived from microalgae and seafood, has been shown to exhibit anti-inflammatory and anti-fibrotic effects through various mechanisms, including the inhibition of transforming growth factor-β1 (TGF-β1) signaling pathways [12,13].

The observed improvement in mouth opening supports the hypothesis that astaxanthin may mitigate the fibrotic processes underlying OSMF. TGF-β1 plays a pivotal role in fibrosis, promoting the deposition of extracellular matrix proteins [14]. Astaxanthin's potential to modulate TGF-β1 signaling may contribute to the attenuation of fibrosis, consequently leading to enhanced mouth opening in individuals with OSMF [9]. Moreover, the twice-daily dosing regimen of astaxanthin was chosen deliberately to maintain sustained levels of the compound in the participants' system. This approach is supported by studies suggesting the importance of continuous exposure to antioxidants for optimal anti-fibrotic effects [15,16].

The reduction in burning sensation, as assessed by the Visual Analog Scale (VAS), further supports the potential therapeutic benefits of astaxanthin in OSMF. The experimental group reported a significant decrease in burning sensation compared to the control group. This outcome is consistent with the antioxidative and anti-inflammatory properties attributed to astaxanthin [17]. The mechanism behind the reduction in burning sensation may be linked to astaxanthin's ability to quench reactive oxygen species (ROS) and modulate inflammatory pathways [18]. OSMF is associated with oxidative stress and inflammation, and astaxanthin's multifaceted antioxidant and anti-inflammatory actions could contribute to ameliorating these symptoms. The VAS results corroborate findings from studies highlighting astaxanthin's potential to mitigate oxidative stress and inflammation in various tissues [10].

The study's emphasis on safety is crucial in evaluating the feasibility of incorporating astaxanthin into OSMF management. Adverse events were generally mild, with the most commonly reported being gastrointestinal upset. These findings align with the established safety profile of astaxanthin, with previous studies reporting minimal adverse effects [19]. The slightly higher incidence of adverse events in the experimental group warrants consideration. However, it's essential to note that the adverse events reported were mild and expected, indicating a generally favorable safety profile. The placebo-controlled design of the study enhances the reliability of these safety assessments, as any observed differences can be attributed to the intervention rather than non-specific factors.

Limitations

While the study provides promising insights into the potential benefits of astaxanthin in OSMF management, several limitations should be acknowledged. The relatively short duration of the intervention (12 weeks) limits the assessment of long-term effects and durability of the observed improvements. Future studies with extended follow-up periods are warranted to elucidate the sustainability of astaxanthin's effects. Moreover, the sample size of 68 participants, although carefully selected, may restrict the generalizability of the findings. Larger-scale trials encompassing diverse demographic groups could provide a more comprehensive understanding of astaxanthin's efficacy across different populations. Incorporating additional outcome measures, such as histopathological assessments of oral mucosal tissue and biomarkers of fibrosis, could offer deeper insights into the underlying mechanisms of astaxanthin's effects. These measures would provide a more comprehensive understanding of the impact of astaxanthin on the histological and molecular aspects of OSMF.

## Conclusions

In conclusion, this randomized, double-blind, placebo-controlled trial suggests that astaxanthin holds promise as a therapeutic intervention in the management of oral submucous fibrosis. The observed improvements in mouth opening and reduction in burning sensation in the experimental group underscore the potential anti-fibrotic, antioxidative, and anti-inflammatory properties of astaxanthin. The safety and tolerability profile observed in this study further supports its feasibility as a therapeutic adjunct. While these findings are encouraging, they warrant further validation through larger, longer-term clinical trials. Future research should explore optimal dosing regimens, potential synergies with existing treatments, and the mechanistic underpinnings of astaxanthin's effects. Astaxanthin's potential to modulate fibrotic pathways in OSMF offers a novel avenue for therapeutic exploration, providing hope for improved outcomes and enhanced quality of life for individuals affected by this challenging condition.
